# Unravelling the effects of nano SiO_2_, nano TiO_2_ and their nanocomposites on *Zea mays* L. growth and soil health

**DOI:** 10.1038/s41598-024-61456-x

**Published:** 2024-06-18

**Authors:** Kusum Kumari, Neelam Rani, Vinita Hooda

**Affiliations:** Department of Botany, Maharshi Dayanand University, Rohtak, 124001 India

**Keywords:** Maize, Nanoparticles, N-fixing bacteria, Nutrient uptake, Phosphate solubilizing bacteria, Silica, Titania, Biochemistry, Physiology

## Abstract

Amidst the challenges posed by climate change, exploring advanced technologies like nanotechnology is crucial for enhancing agricultural productivity and food security. Consequently, this study investigated the impact of nano SiO_2_ (nSiO_2_), nano TiO_2_ (nTiO_2_) and SiO_2_/TiO_2_ nanocomposites (NCs) on 30-day-old *Zea mays* L. plants and soil health at concentrations of 100 and 200 ppm. Results showed that nSiO_2_ and nTiO_2_ at 100 ppm and SiO_2_/TiO_2_ NCs at both concentrations, positively influenced plant growth, with the best stimulation observed at 200 ppm of SiO_2_/TiO_2_ NCs. Improved plant growth was associated with higher chlorophyll content, photosynthetic rate, transpiration rate, stomatal conductance, rhizospheric N-fixing and phosphate solubilizing bacterial population and plant nutrient uptake. Additionally, treated plants exhibited increased cellulose and starch levels. Malondialdehyde (MDA) content was lower or similar to that of the control, except at 200 ppm of nTiO_2_-treated shoots. Antioxidant enzyme activities fluctuated, indicating physiological adjustments. Overall, 100 ppm of nTiO_2_ as well as nSiO_2_ and 100 and 200 ppm of SiO_2_/TiO_2_ NCs improved soil fertility and *Z. mays* growth, suggesting potential benefits for sustainable agriculture. The findings lay the foundation for more comprehensive investigations into the long-term fate of nanomaterials in soil and their intricate molecular-level interactions with *Z. mays*.

## Introduction

Agriculture significantly contributes to climate change due to its high energy consumption and reliance on consumerism. Pesticides and fertilizers used in agriculture to increase production are major sources of pollution, contaminating water sources and causing soil erosion^1^. Polluted agricultural soils have a negative impact on yield, human well-being and economies. Moreover, the global population is expected to reach 10 billion by 2050 and the zero hunger goal of the United Nations needs to be achieved while also addressing environmental concerns and ensuring sustainable agricultural practices. *Zea mays* L. (maize), a highly valued cereal crop with a relatively short growing time and a high yield makes a staple food of about one-third of the world’s population, mainly in the developing world^2^. Sustainable production of *Z. mays* in the face of climate change, rising food demand and decreased soil fertility is therefore critical for ensuring a stable food supply.

Nanotechnology is a new strategy that supports sustainable agriculture while increasing crop productivity. Nanomaterials can alleviate agricultural and environmental problems by acting as adsorbents, catalysts, carrier systems, growth-promoting and antibacterial substances^3^. The type of nanomaterial and its properties such as its shape, size, surface chemistry and concentration affect its interaction with the plants and the surrounding environment, which may in turn impact plant growth and productivity^4^.

In this respect, titanium dioxide nanoparticles (nTiO_2_) have shown promising results and even outperform metallic nanoparticles that are unstable in water^5^. The nTiO_2_-based materials have been shown to achieve in-situ detoxification of hazardous agricultural pollutants during photocatalysis and promote mineralization, which reduces heavy metal bioaccumulation. nTiO_2_ improves the utilization of light energy for photosynthesis, thus, the photosynthetic efficiency, which promotes plant growth. They also have the ability to modify soil properties, including improving soil aeration and moisture retention, which improves soil structure and increases plant nutrient availability. Loadings of nTiO_2_ less than 10 g L^−1^ and sizes smaller than 40 nm improved seed germination and root elongation while minimizing metal root translocation in maize^6^. Hassan et al.^7^ demonstrated that nTiO_2_ positively impacted the shoot length, chlorophyll levels and antioxidant enzyme activities in sorghum. Additionally, at a concentration of 100 mg L^−1^, nTiO_2_ led to increased growth in *Dracocephalum moldavica*, as evidenced by enhanced plant height, leaf number and fresh and dry weight of the treated plants^8^.

On the contrary, several studies also suggest the noxious nature of nTiO_2_, primarily attributed to its ability to generate reactive oxygen species (ROS) within plant cells. Excessive exposure to nTiO_2_ may also disrupt the balance of essential nutrients in plants. In maize, seed germination decreased after priming with nTiO_2_ at three distinct concentrations (250, 500, and 1000 µg mL^−1^)^9^.

Nano-silica (nSiO_2_), a natural component of soil, is used in agriculture because of its excellent properties, such as no toxicity, low cost, high surface area, better biocompatibility and tunable pore volume. Since it is a natural component of the earth’s crust, all rooted plants accumulate silica in their tissues to provide structural support^10^. The application of nSiO_2_ is known to accelerate plant growth by stimulating photosynthesis and mitigating oxidative stress^11^. In some rice crop trials, significant dosages of silica (960 kg ha^−1^) were utilized because bulk silica can occasionally be unavailable to plants due to polymerization activities in the soil that turn silica into a gel form^12^. As nSiO_2_ are mesoporous and typically very small in size, they are absorbed more effectively^13,14^ and hence compared to bulk silica, fewer doses of nSiO_2_ are applied to the soil while keeping the efficacy. Silica accumulation in *Z. mays* treated with nSiO_2_ was 9.14% more than in plants treated with bulk silica^15^. Though most studies have found nSiO_2_ to be beneficial for plants^16^, in some cases they may also exert negative effects. Ghoto et al.^17^ reported that nSiO_2_ significantly decreased the growth of 6-day-old maize plants at 1000 mg L^−1^ concentration. The negative surface charge and low zeta potential of nSiO_2_ slow down their contact with plants and cause them to chelate with other minerals, including Cu, Zn, Mo and Mn, which causes plants to be deficient in micronutrients^18^.

The use of nanocomposites (NCs) made by combining metals with other inorganic or organic materials has gained attention because of their superior qualities over individual nanoparticles^19^. An NC of nSiO_2_ and nTiO_2_ is expected to exhibit synergistic effects and improved performance as it will combine the biocompatible highly porous structure and large specific surface area of nSiO_2_ with the photocatalytic activity of nTiO_2_. Adding positively charged nTiO_2_ to nSiO_2_ may also limit excessive chelation of other nutrients by Si while providing adequate positive surface charge to improve interactions with plant cells. Moreover, the complexation of nTiO_2_ with nSiO_2_ will decrease the TiO_2_ fraction available to produce hazardous metal ions and hence damaging ROS.

Though the effects of nSiO_2_ and nTiO_2_ on *Z. mays* have been the subject of multiple research, the findings have been equivocal and there are still significant gaps in our understanding at biochemical and physiological levels. Furthermore, the impact of SiO_2_/TiO_2_ NCs on *Z. mays* has not yet been investigated. Therefore, the current work assesses the morphological, biochemical, and physiological changes in 30-day-old *Z. mays* plants cultivated in nSiO_2_, nTiO_2_ and SiO_2_/TiO_2_ NCs spiked soil. Additionally, the effects of nSiO_2_, nTiO_2_ and SiO_2_/TiO_2_ NCs on plant nutrient uptake and soil microflora have also been tested as a marker of soil health.

## Materials and methods

### Chemicals

All chemicals utilized were of AR grade and procured from Hi-Media Laboratories Pvt. Ltd., Mumbai; Sigma-Aldrich Chemicals Pvt. Ltd., Mumbai; Moly Chem, Mumbai; Merck Life Science Pvt. Ltd., Mumbai; Thomas Baker Chemicals Pvt. Ltd, Mumbai; Loba Chemie Pvt. Ltd. Mumbai.

### Synthesis of nSiO_2_

The Stober technique^20^ was used to synthesize nSiO_2_. To make nSiO_2_, tetraethoxysilane (TEOS) (11.2 mL) was added to a mixture of 10 mL ethanol and 35 mL deionized water and stirred for 10 min. After that, some drops of hydrochloric acid (HCl) (1 M) were added and the solution was stirred magnetically (Tarsons-SPINOT DIGITAL, MC02, India) for 50 min at 60 °C until it became a homogenous white translucent solution. The prepared solution was left at 25 °C for 2 h to facilitate gel formation. After 6 h of drying at 90 °C in an oven, crystalline SiO_2_ particles were ground to yield a white powder of nSiO_2_.

### Synthesis of nTiO_2_

The nTiO_2_ were prepared by the procedure followed by Ghows and Entezari^21^. A mixture of glacial acetic acid (0.2 mL) and distilled water (50 mL) was sonicated (Q-Sonica Sonicators, Q125, U.S.A.) for 10 min. To it, a solution of 2 mL titanium tetra-isopropoxide (TTIP) and 5 mL ethanol was added dropwise and sonicated at 25 °C for 3 h (20 kHz,125 W, 70% amplitude). After centrifuging (Plasto Crafts, Rota 4R-V/Fm, India) the solution at 17,608×*g* for 20 min, the precipitates were repeatedly cleaned with distilled water and ethanol. The product was dried for 6 h at 40 °C.

### Synthesis of SiO_2_/TiO_2_ NCs

TEOS and TTIP were selected as the sources of silica and titania, respectively^22^. NCs were synthesized in two steps. In the first step, under constant stirring, TEOS was added dropwise to the methanol and both were combined in 1:6 ratios to synthesize nSiO_2_. Then, 0.05 mol (1.52 mL) HCl was added dropwise to the solution to maintain a pH of 2.0. The solution was stirred for around 2 h to get a homogenous solution. In the second step, TTIP and distilled water were mixed in a 1:14 ratio until a homogenous TiO_2_ solution was formed. Then, TiO_2_ and SiO_2_ were mixed in a 1:1 ratio under continuous stirring until a homogenous solution was formed and to it, 0.05 mol (2.2 mL) NH_4_OH solution was added dropwise. Finally, the mixture was stirred for 10 min and dried at 90 °C to get the SiO_2_/TiO_2_ NCs powder.

### Characterization of Nanomaterials

Transmission electron microscopy (TEM; Hitachi, H-7500, 120 kV) was used to determine the size of the nanomaterials, field emission scanning electron microscopy (FESEM; Hitachi, SU8010 Series) was used to confirm their morphology and X-ray diffraction (XRD; Panalytical X’Pert Pro) was used to determine crystal structure of nanomaterials at Sophisticated Analytical Instrument Facility (SAIF), Punjab University, Chandigarh. The TEM images were examined using the free Image J program (https://imagej.nih.gov/ij/) to determine the size of nanoparticles and NCs. Hydrodynamic diameter, zeta potential and the polydispersity index (PDI) of the synthesized nanomaterials were measured using a Zetasizer analyzer (Malvern Panalytical, Version 7.13) at Central Instrumentation Laboratory, Maharshi Dayanand University, Rohtak.

### Experimental design

The seeds of *Z. mays* (HQPM-7) were purchased from the Regional research centre of Haryana Agriculture University at Uchani, Karnal. The seeds were procured and used by following all the relevant guidelines. They were immersed in a 0.1% mercuric chloride solution for 2 min to ensure surface sterilization and rinsed at least 5 times with distilled water. Fertile farm soil was collected from the Research Centre, MDU, Rohtak and air-dried before experimental use. Soil analysis by ICP-MS (ICAP 600 Series, ICP Spectrometer from Thermo Scientific) at Soil Testing Lab, Rohtak revealed that it had the following properties: Electrical conductivity 0.23 dS m^−1^, organic carbon 0.54%, pH 7.02, Zn 3.14 ppm, Fe 19.54 ppm, Mn 4.04 ppm, S 15.32 ppm, K 65 ppm and P 14 ppm.

A total of seven experimental groups were raised in pots: one control group that had no nanomaterial at all and six treatment groups, corresponding to plants raised in soil spiked with either nSiO_2_ or nTiO_2_ or SiO_2_/TiO_2_ NCs at two different concentrations i.e., 100 and 200 ppm, respectively. Each group had five replicates with five plants per replicate, making a total of 25 plants per experimental group. The emergence of the radicle was considered an indicator of seed germination. The plants were grown at the average day/night temperature of 37.0/21.8 °C. They received photosynthetic photon flux density of 1480 to 1850 µmol m^2^s^−1^ light intensity on a clear, bright sunny day in April/May, having approximately 12 h light and 12 h dark period.

Whole plants were carefully removed from the soil after 30 days. Rhizospheric soil samples were collected from plant roots and the plants were carefully washed with distilled water. The roots and shoots were separated and their length, fresh and dry weight were measured. The number of axial roots was also counted. For further investigation, the plants were crushed in liquid nitrogen and stored at − 20 °C. The collected rhizospheric soil was air dried and utilized for checking the plant growth-promoting bacteria by serial dilution method.

### Mineral and metal uptake by *Z. mays* plants

The shoots and roots of the harvested plant samples were dried at 80 °C for 48 h to examine the amount of mineral nutrients (N, P, K and Mg) taken up by control and treated *Z. mays* plants^23^. The contents of Si and Ti in dried plant samples were also analyzed by Energy Dispersive Spectroscopy (EDS, Hitachi: 8010) at SAIF, Punjab University, Chandigarh.

### Chlorophyll and protein content

The determination of chlorophyll contents was based on the method of Thakur et al.^24^. Protein contents of the root and shoots were measured by the Bradford method^25^ using the standard curve of BSA (10–100 mg g^−1^). The results for both chlorophyll and protein were expressed as mg g^−1^ of plant material.

### Starch and cellulose content

To measure starch content, the plant sample (0.1 g) was dissolved in 10 mL hot ethanol (80%) to remove all the sugars and then 6.5 mL perchloric acid (52%) was added to convert starch to glucose units^26^. The amount of glucose was estimated by the phenol method. To determine cellulose content, the plant sample (0.1 g) was digested in 6 mL of nitric acid. After digestion, all sugars were removed except cellulose and further, anthrone assay was used for cellulose quantification^27^. Starch and cellulose contents were calculated using a glucose standard curve (10–100 µg mL^−1^) and results were represented in mg g^−1^ of plant material.

### Gas exchange parameters

In 30-day-old nanomaterials treated and control plants, gas exchange parameters such as net photosynthesis (A), stomatal conductance (G) and transpiration rate (E) were measured with a handheld gas exchange device (LCi-SD, ADC BioScientific Ltd., UK). Observations were made on fully inflated leaves between 10:00 and 12:00 A.M. at 26 ± 0.1 °C. At the time of measurement, CO_2_ concentration was 300 ppm and the photon flux density was 1250 ± 10 µmol m^−2^ s^−1^^28^.

### Lipid peroxidation and antioxidant enzymes activities

Heath and Packers^29^ method was used to calculate the amount of MDA produced by lipid peroxidation, which was reported as µM MDA g^−1^ fresh weight. The present work investigated the nSiO_2_, nTiO_2_ and SiO_2_/TiO_2_ NCs-induced modulations in the activities of four antioxidant enzymes: superoxide dismutase (SOD), catalase (CAT), ascorbate peroxidase (APx), and glutathione peroxidase (GPx). To accomplish this, 0.5 g of plant tissue was homogenized in 5 mL of potassium phosphate buffer (0.1 M, pH 7.0) and subsequently cold centrifuged for 10 min at 17,608×*g*. The resulting supernatant served as the enzyme source for subsequent experiments. In all experiments, potassium phosphate buffer (0.1 M, pH 7.0) was used as the blank.

SODs are metalloproteins that catalyze the dismutation reaction of superoxide to O_2_ and H_2_O_2._ This assay was performed by NBT (nitroblue tetrazolium) reduction method^30^. CAT activity was proportional to a decrease in A_240_ nm due to the decomposition of H_2_O_2_ into H_2_O and O_2_^31^ and the unit activity of CAT was expressed as U mg^−1^ min^−1^. APx activity was calculated by detecting the decrease of A_290_ nm due to oxidation of ascorbate by H_2_O_2_^32^. The activity of GPx was directly related to the quantity of oxidized glutathione and was assessed using the procedure described by Rani et al.^33^.

### Influence on soil microorganisms

The effects of nanomaterials on soil microbes were determined by culturing N-fixers and phosphate solubilizers on their specific media. *Azotobacter* and *Azospirillum* are free-living, gram-negative and aerobic N-fixers that were isolated using Ashby’s mannitol media and *Azospirillum* medium with 0.17% agar, respectively. Pikovaskay’s growth media was used to check the growth of phosphate solubilizers.

### Statistical analysis

A completely randomized block design was used in the experiment. The experimental data was analyzed using IBM SPSS Statistics 26 software for one-way ANOVA and a post-hoc analysis with the Tukey test. Different letters represent significant differences at *p* ≤ 0.05. Origin 8 (Origin Lab Corporation, United States) was used to prepare graphs. Standard error bars in the graph indicate the variability of data.

## Results and discussion

### Characterization

**Figure 1:**
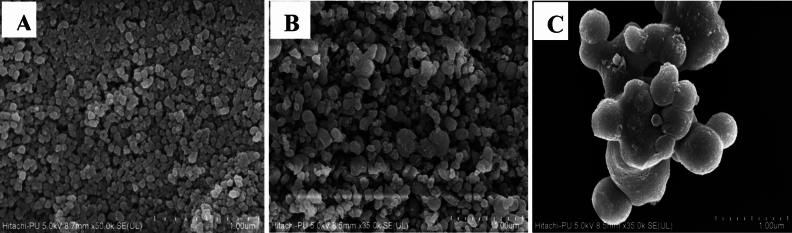
FESEM images of nSiO_2_ (**A**), nTiO_2_ (**B**) and SiO_2_/TiO_2_ NCs (**C**).

**Figure 2:**
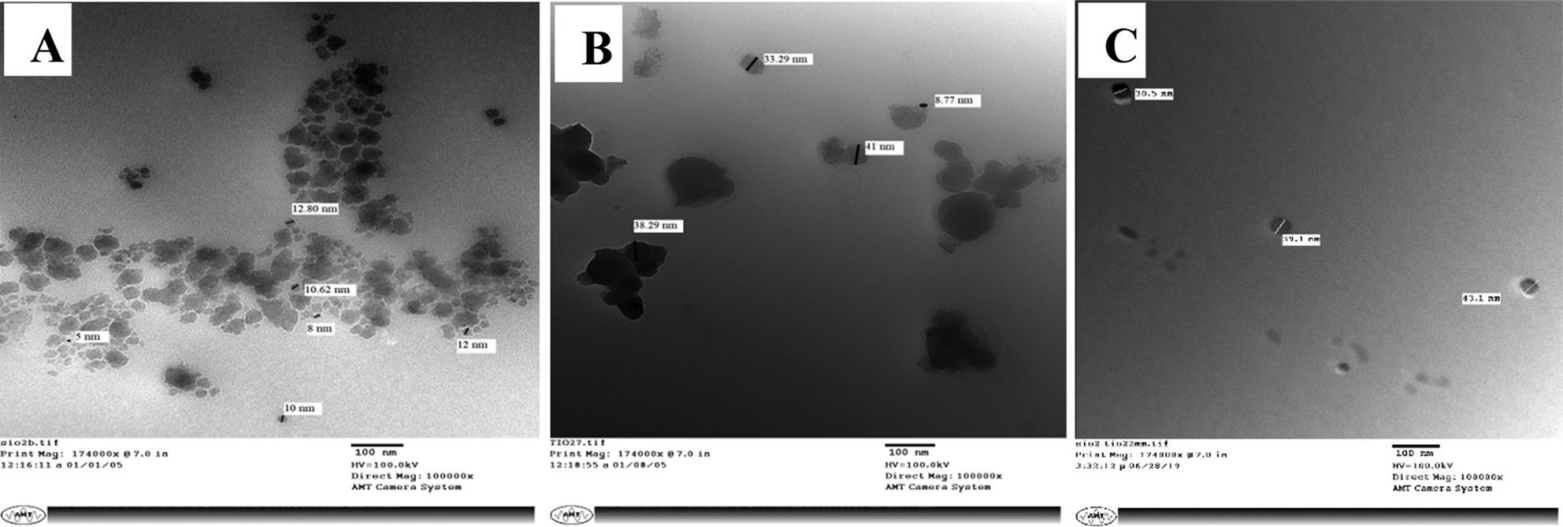
TEM images of nSiO_2_ (**A**), nTiO_2_ (**B**) and SiO_2_/TiO_2_ NCs (**C**).

FESEM images of the nSiO_2_ (Fig. 1A) and nTiO_2_ (Fig. 1B) indicated that they were spherical to polygonal in shape. The nSiO_2_ were well dispersed, while the nTiO_2_ showed a propensity to aggregate. The network of irregular spheres is shown to be interconnected and grouped in the SiO_2_/TiO_2_ NCs micrograph shown in Fig. 1C. The TEM picture of nSiO_2_, as seen in Fig. 2A, revealed that the size of the nanoparticles ranged between 5 and 10 nm. The nTiO_2_ were clustered with an average size of 20–30 nm (Fig. 2B), whereas in Fig. 2C, the TEM image of SiO_2_/TiO_2_ NCs indicated that the size of NCs was between 30 and 40 nm.

The hydrodynamic diameter of nSiO_2_, nTiO_2_ and SiO_2_/TiO_2_ NCs suspension as determined by Zetasizer analyzer was 256.6 ± 78.36 nm, 369.1 ± 138.7 nm and 375.5 ± 145.8 nm and zeta potential values were − 25.4 ± 4.10, 38.2 ± 4.96 and − 4.09 ± 3.63 mV respectively Supplementary Fig. 1). According to zeta potential values, nSiO_2_ were stable, nTiO_2_ were highly stable and NCs were unstable. The positive charge of nTiO_2_ decreased the negative charge of nSiO_2_ after combining with it. The presence of a negative charge on the NCs surface suggests that negatively charged nSiO_2_ were present on the surface and nTiO_2_ was present in the core of the NCs.

The size distribution of nanoparticles can be explained by the polydispersity index (PDI) value. The PDI values for the nSiO_2_, nTiO_2_, and SiO_2_/TiO_2_ NCs suspension in the current investigation were 0.54 ± 0.02 (highly polydispersed), 0.208 ± 0.01 (moderately dispersed) and 0.355 ± 0.03 (moderately dispersed), respectively. Results indicated that the moderately dispersed suspension produced by the NCs had a better size distribution than that of the highly polydisperse nSiO_2_ suspension.

**Figure 3:**
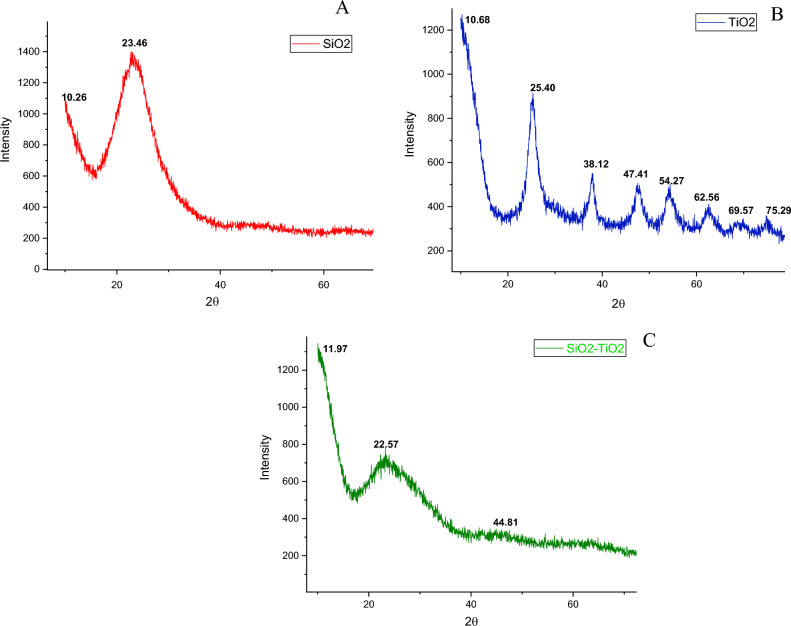
XRD pattern of nSiO_2_ (**A**), nTiO_2_ (**B**) and SiO_2_/TiO_2_ NCs (**C**).

The XRD pattern of nSiO_2_, nTiO_2_ and the NCs is given in Fig. 3. The XRD pattern of nSiO_2_ showed the presence of a very broad peak displayed around 18–25 2Ѳ (Fig. 3A). This broad peak confirmed the small size and amorphous nature of the synthesized nanoparticles^34^. The nSiO_2_ did not contain any additional impurity peaks. XRD pattern of nTiO_2_ in Fig. 3B, showed sharp peaks at 25, 38, 47, 54 and 62 thus confirming the crystalline phase of nTiO_2_, which is quite similar to the nTiO_2_ reference pattern (JCPDS 21-1272). The broad peak visible in Fig. 3C, which is located around 20–30 2Ѳ, indicated that the SiO_2_/TiO_2_ NCs were amorphous in character. Xie et al.^35^ reported that the addition of a small amount of nSiO_2_ into pure nTiO_2_ inhibits the growth of crystalline structure.

### Soil analysis

Electrical conductivity (EC = 0.23 dS m^−1^), organic carbon (OC = 0.54%), pH (7.02, Neutral) and nutrients: Zn (3.14 ppm), Fe (19.54 ppm), Mn (4.04 ppm), S (15.32 ppm), K (65 ppm) and P (14 ppm) present in soil were analyzed by ICP-MS at Soil Testing Lab, Rohtak. All the nutrients were present in the soil in between the quantity suggested by the agriculture and farmer welfare department report, Haryana. According to the soil analysis results, the soil quality was good for conducting the experiments. Soil organic matter, in particular, has the potential to alter NP mobility and bioavailability by influencing their aggregation state, surface charges, polarization state, zeta potential and intermolecular interactions^36^.

### Quantification of nanomaterials

**Figure 4:**
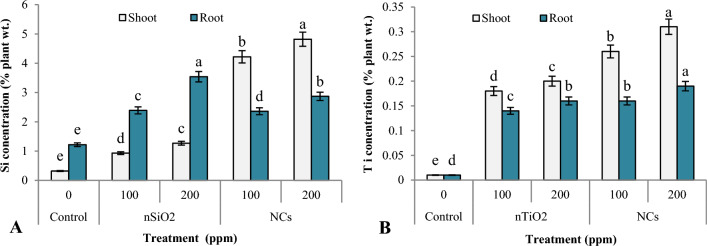
Accumulation of Si (**A**) and Ti (**B**) in the shoots and roots of 30-day-old *Z. mays* plants, as determined by EDS. Data represents the mean of five replicates, with standard error bars indicating the variability of data.

Dried plant samples were analyzed by EDX for quantification of nanoparticles accumulated by them. Percent accumulation of Si in control and nSiO_2_-treated plants at 100 and 200 ppm concentration was found to be 0.32% in control and 0.93 and 1.27%, respectively (Fig. 4A). In the roots, too, percent Si accumulation increased from 1.22 in control to 2.39 and 3.54% at 100 and 200 ppm, respectively. Suriyaprabha et al.^37^ also reported that 10 and 15 kg ha^−1^ nSiO_2_ increased Si accumulation in maize plants by 0.57 and 0.82%, respectively. In SiO_2_/TiO_2_ NCs treated plants, there was approximately 4–5 times more percent accumulation of Si in the shoots (i.e. 4.22% at 100 ppm and 4.82% at 200 ppm concentration) compared to the control, while the percent accumulation of Si in the roots of NCs was 2.36% at 100 ppm and 2.87% at 200 ppm concentration. When plants were treated with nSiO_2_, the accumulation of Si in the roots was higher compared to the shoot, whereas for NCs treated plants, the shoots accumulated more Si than the roots. Si is abundant in the soil and the charge on nanoparticles, plant roots, cellular components, biomolecules and cell wall influences its uptake and transportation in plants. The nSiO_2_ having higher negative charge intensity is not well retained by the negatively charged cell wall; instead, it interacts strongly with other charged plant cell constituents like proteins and nucleic acids, which may impede its passage through plasmodesmata and limit its ability to migrate from one cell to another. Furthermore, the Casparian strip also prevents negatively charged nSiO_2_ from moving radially^38,39^. Conversely, the presence of fewer negative charges on the surface of NCs in comparison to nSiO_2_ may have facilitated their symplastic transportation to the central stele and subsequent translocation to the above-ground parts of the plant^40,41^. Additionally, in SiO_2_/TiO_2_ NCs, nTiO_2_ interacts with the nSiO_2_, imparting it a hydrophilic character that may favour their adsorptive behaviour in soil^42^.

Titanium (Ti) accumulation in the shoots of *Z. mays* plants increased from 0.02% in the control to 0.18 and 0.20% in the nTiO_2_ treatments at 100 and 200 ppm concentrations, respectively (Fig. 4B). Ti accumulation in the roots increased from 0.02% in the control to 0.14% and 0.18% at 100 and 200 ppm, respectively. Similar Ti accumulation was found by Thakur et al.^24^ in mung bean plants treated with nTiO_2_ at doses of 10 and 100 mg L^−1^. As shown in Fig. 4B, the percent accumulation of Ti in the shoots was higher in NCs (0.26% at 100 and 0.31% at 200 ppm concentration) than in nTiO_2_. The matrix composition, which includes the amount of organic matter, free ions and pH, influences TiO_2_ mobility in soils^43^. Additionally, changes in the surface area, zeta potential and surface affinity may all affect TiO_2_ availability in soil and plants^44^.

In the nTiO_2_-treated plants, the accumulation of Ti in the shoots was lower compared to the Ti content in the shoots of NC-treated plants. Once the metal ion is absorbed, its accumulation in the shoots is directly related to the ability of plants to transport it from cell to cell. Because of the higher positive charge on the nTiO_2_, they might have interacted more strongly with the negatively charged cell wall and other biomolecules in the cytoplasm, hindering their movement both through the apoplast and symplast and hence their accumulation in the shoots.

### Effect on growth parameters

The effect of nSiO_2_, nTiO_2_ and SiO_2_/TiO_2_ NCs on the growth parameters of *Z. mays* after 30 days of growth is presented in Table 1. The shoot length of nSiO_2-_treated plants was comparable to control plants at 100 ppm while it decreased by 12% at 200 ppm. The root length increased by 20% at 100 ppm of nSiO_2_ and decreased marginally by 4% at 200 ppm. Both concentrations of nSiO_2_ increased parameters such as root and shoot number, as well as fresh and dry weight, however, the stimulation was higher at 100 ppm compared to 200 ppm. The fresh weight of the shoot and root increased by 9 and 41% and the dry weight by 49 and 5% at 100 ppm, respectively. At 200 ppm of nSiO_2_, the fresh and dry weight of the shoot and root increased slightly and were largely comparable to the control plants. nTiO_2_ at 100 ppm stimulated shoot growth, with a slight increase of 2% in shoot length, 5% fresh weight and a substantial increase of 52% in dry weight, respectively. However, a slight inhibition of shoot growth was observed at 200 ppm, with decreases of 4%, 10%, and 24% in shoot length, fresh weight, and dry weight, respectively. Contrary to this, both 100 and 200 ppm concentrations of nTiO_2_ stimulated root growth, however, the growth at 200 ppm was lower compared to 100 ppm concentration. The root length increased by 9 and 4%, root fresh weight by 28 and 12% and root dry weight by 22 and 8% at 100 and 200 ppm of nTiO_2_. As evident from Table 1, NCs of SiO_2_/TiO_2_ increased all the parameters in a concentration-dependent manner in *Z. mays* plants. NCs increased the shoot length by 2 and 6%, root length by 3 and 23%, shoot fresh weight by 9 and 12%, root fresh weight by 1 and 50%, shoot dry weight by 7 and 31% and root dry weight by 2 and 5% at 100 and 200 ppm respectively.

**Table 1:** Effect of nSiO_2_, nTiO_2_ and SiO_2_/TiO_2_ NCs on the growth of 30-day-old *Z. mays* plants. Data is presented as mean ± S.D (n = 5), with different letters indicating significant differences at *p* < 0.05.

Treatments	Concentrations (ppm)	Shoot length (cm)	Root length (cm)	Number of roots	Fresh weight(g)		Dry weight(g)	
					Shoot	Root	Shoot	Root
Control	00	61.86 ± 0.12^bc^	36.1 ± 0.74^e^	11. ± 0.44^e^	16.25 ± 0.10^c^	4.51 ± 0.25^d^	2.23 ± 0.24^c^	0.88 ± 0.03^c^
nSiO_2_	100	62.54 ± 0.41^abc^	43.4 ± 0.38^b^	13.6 ± 0.54^ab^	17.76 ± 0.21^a^	6.36 ± 0.18^a^	3.31 ± 0.15^ab^	0.92 ± 0.08^bc^
	200	54.48 ± 0.85^d^	34.48 ± 0.86f.	12.2 ± 0.83^d^	16.56 ± 0.21^c^	4.63 ± 0.27^d^	2.43 ± 0.18^c^	0.89 ± 0.03^c^
nTiO_2_	100	63.22 ± 0.30^ab^	39.52 ± 0.35^c^	13.2 ± 0.44^bc^	17.14 ± 0.47^b^	5.77 ± 0.12^b^	3.4 ± 0.37^a^	1.07 ± 0.02^a^
	200	59.28 ± 0.52^c^	37.63 ± 0.40^d^	12.8 ± 0.44^c^	14.63 ± 0.27^d^	5.06 ± 0.01^c^	1.70 ± 0.14^d^	0.94 ± 0.01^b^
NCs	100	63.14 ± 0.53^ab^	37.21 ± 0.17^d^	13 ± 0.0^bc^	17.67 ± 0.21^a^	4.53 ± 0.24^d^	2.38 ± 0.13^c^	0.89 ± 0.02^c^
	200	65.72 ± 0.79^a^	44.56 ± 0.20^a^	14 ± 0.0^a^	18.17 ± 0.10^a^	6.75 ± 0.18^a^	2.91 ± 0.03^b^	0.92 ± 0.03^bc^

The nSiO_2_ promotes root mass and development, enabling plants to penetrate the soil and facilitating the absorption of water and nutrients. Liu et al.^45^ and Liu and Lal^46^ also reported that nSiO_2_ increased germination percentage, lateral root development, and plant biomass in rice plants. Though nSiO_2_ is largely associated with improved plant growth, at higher concentrations it may potentially interfere with the absorption of other essential nutrients including N and P^47^. Slightly reduced shoot and root length by nSiO_2_ at 200 ppm could possibly be due to reduced absorption of N and P by the roots of these plants (Table 3).

The nTiO_2_ may promote cell expansion and plant growth by loosening the cell wall and increasing membrane fluidity^48^. Growth stimulation by nTiO_2_ is also in part due to the enhanced ability of plants to absorb nutrients from the soil^49^, as observed in the present study (Table 3). Nonetheless, at higher concentrations, nTiO_2_ also produces damaging ROS, which inhibits the growth of plants. Stimulatory effects of nTiO_2_ (100 mg L^−1^) on roots were previously demonstrated in wheat^49^ and in chickpea^50^ plants. Zahra et al.^51^ reported that root growth and development was influenced by the absorption of nutrients like K, N, Mg and P by the plants in the presence of nTiO_2_. Raliya et al.^52^ also concluded that nTiO_2_-induced uptake of mineral elements in the plants led to increased growth and biomass of mung bean plants. In another study, it was observed that nTiO_2_ had a positive impact on wheat at concentrations up to 60 mg kg^−1^, but at higher levels (100 mg kg^−1^), it had an adverse effect on wheat roots^53^.

Enhanced growth by the NCs might be due to increased uptake of N, P and K by the SiO_2_/TiO_2_ NCs treated plants (Table 3) which besides providing essential micronutrients also enhance their abilities to absorb and utilize water resulting in enhanced growth.

### Photosynthetic parameters

Total chlorophyll content, photosynthetic rate, transpiration rate and stomatal conductance in 30 days old *Z. mays* plants at both 100 and 200 ppm of nSiO_2_, nTiO_2_ and SiO_2_/TiO_2_ NCs are presented in Table 2. Chlorophyll content increased by 75 and 29% in nSiO_2_ treated plants, 66 and 79% in nTiO_2_ treated plants and 34 and 81% in NCs treated plants at 100 and 200 ppm concentrations, respectively. The percentage increase in transpiration rate was 68, 24 and 87% at 100 ppm and 4, 101 and 141% at 200 ppm of nSiO_2_, nTiO_2_ and NCs, respectively. Stomatal conductance increased by 67, 33 and 100% at 100 ppm for nSiO_2_, nTiO_2_ and NCs, respectively, whereas at 200 ppm, it increased for nTiO_2_ and NCs-treated plants by 150 and 200% and decreased for nSiO_2_ treated plants by 17%_._

**Table 2:** Effect of nSiO_2_, nTiO_2_ and their NCs on photosynthetic efficiency, starch and cellulose contents in 30-day-old *Z. mays* plants. Data is presented as mean ± S.D (n = 5), with different letters indicating significant differences at *p* < 0.05.

	Control	nSiO_2_		nTiO_2_		NCs	
	00 ppm	100 ppm	200 ppm	100 ppm	200 ppm	100 ppm	200 ppm
Chl (mg g ^- 1^)	5.07 ± 0.29^c^	8.89 ± 0.45^a^	6.55 ± 0.43^b^	8.42 ± 0.07^a^	9.08 ± 0.27^a^	6.77 ± 0.54^b^	9.17 ± 0.40^a^
A (µmol CO_2_ m^−2^ s^−1^)	11.25 ± 0.27^e^	17.17 ± 0.15^a^	11.96 ± 0.64^de^	12.6 ± 0.18^cd^	15.0 ± 0.94^b^	12.8 ± 0.22^c^	13.6 ± 0.31^c^
E (mmol H_2_O m^-2^ s^−1^)	1.43 ± 0.18^b^	2.4 ± 0.21^ab^	1.48 ± 0.16^b^	1.77 ± 0.10^ab^	2.87 ± 0.78^ab^	2.67 ± 0.59^ab^	3.45 ± 1.43^a^
G (µmol CO_2_ m^-2^ s^−1^)	0.02 ± 0^b^	0.03 ± 0.005^ab^	0.017 ± 0.01^b^	0.027 ± 0.01^ab^	0.05 ± 0.02^ab^	0.04 ± 0^ab^	0.06 ± 0.02^a^
St-S (mg g^−1^)	0.055 ± 0.44^ g^	0.078 ± 0.55^b^	0.064 ± 0.83^e^	0.069 ± 0.54^c^	0.066 ± 0.83^d^	0.063 ± 0.0f.	0.081 ± 0.44^a^
St-R (mg g^−1^)	0.060 ± 0.89^e^	0.089 ± 0.83^b^	0.063 ± 0.70^d^	0.089 ± 0.54^b^	0.081 ± 0.83^c^	0.062 ± 0.54^d^	0.103 ± 0.54^a^
Cel-S (mg g^−1^)	0.003 ± 0.15f.	0.008 ± 0.28^b^	0.011 ± 0.6^a^	0.005 ± 0.37^d^	0.007 ± 0.51^c^	0.004 ± 0.18^e^	0.011 ± 0.40^a^
Cel-R (mg g^−1^)	0.005 ± 0.33^e^	0.007 ± 0.81^bc^	0.006 ± 0.21^cd^	0.006 ± 0.66^de^	0.011 ± 0.7^a^	0.005 ± 0.45^de^	0.007 ± 0.44^b^

A, photosynthetic rate; E, transpiration rate; G, stomatal conductance; Chl, Total chlorophyll; St-S, starch shoot; St-R, starch root; Cel-S, cellulose shoot; Cel-R, cellulose root.

Though, the values of all photosynthetic parameters significantly increased in all treatment groups compared to the control, however, the best stimulation was observed at 200 ppm of SiO_2_/TiO_2_ NCs. This suggests that NCs facilitated increased translocation of Si and Ti to the leaves might have enhanced the photosynthetic capacity of the *Z. mays* plants.

Suriyaprabha et al.^37^ reported that nSiO_2_ led to an increase in Si accumulation, enhancing the photosynthetic activity of *Z. mays* plants by enhancing the leaf surface area. This, in turn, improved light absorption and the photosynthetic activity of both chlorophyll a and b. Similar findings were documented for nTiO_2_ treatment on wheat plants by Shafea et al.^54^. A study on wheat cultivars soaked in various concentrations of nTiO_2_ (0.025, 0.05, 0.1, 0.2, and 0.5%) revealed that 0.1% nTiO_2_ significantly improved seed potential by raising vigour index, plant height and root length, shoot and root dry weight, fresh matter and the composition of photosynthetic pigments such as chlorophyll. Furthermore, nTiO_2_ have been demonstrated to improve plant uptake of many mineral nutrients, including Ca, Mg and K, which improve photosynthetic efficiency by enhancing chlorophyll synthesis and gas exchange^55^. A study conducted by Yang et al.^56^ revealed that in spinach, nTiO_2_ promotes the organic N content of the plant such as the amount of chlorophyll and proteins by increasing the absorption of inorganic N from the soil.

Carbohydrates are formed in plants by photosynthesis and make up about 70% of solid plant material. Additionally, *Z. mays* is a rich source of cellulose, a type of carbohydrate that directly influences growth, mechanical support, the conversion of biomass into fuel and the development of other biomass-based products^57^. Cellulose content was increased by both the nanoparticles and their NCs in both root and shoot at all the concentrations, which correlates well with enhanced chlorophyll content and photosynthetic rate^58^. The percentage increase of cellulose in shoots of treated plants was 124 and 196% for nSiO_2_, 56 and 88% for nTiO_2_ and 23 and 196% for NCs at 100 and 200 ppm concentrations, respectively. In roots, the percentage increase was 32% and 21% for nSiO_2_, 11 and 120% for nTiO_2_ and 2 and 42% for NCs at 100 and 200 ppm concentrations, respectively.

Starch content showed significant enhancement across all treatments. At 100 ppm, there was an approximate increase of 42, 25 and 15% in shoots treated with nSiO_2_, nTiO_2_ and NCs, respectively. Similarly, at 200 ppm, the increase was approximately 16, 20 and 47% for the respective treatments. In roots, there was about a 48% increase at 100 ppm for both nSiO_2_ and nTiO_2_, with a marginal 3% increase for NCs. At 200 ppm concentration, the root starch content increased by 5, 35 and 72% after treatments with nSiO_2_, nTiO_2_ and NCs, respectively (Table 2). The starch content in both the shoots and roots of *Z. mays* was higher at a 100 ppm concentration of both nSiO_2_ and nTiO_2_ compared to the content at 200 ppm of these nanomaterials. However, there was an increase in starch content with the rise in concentration of SiO_2_/TiO_2_ NCs. The increase in starch and cellulose content might be a direct consequence of the enhanced photosynthetic efficiency of the treated plants.

### Protein content and antioxidant status

**Figure 5:**
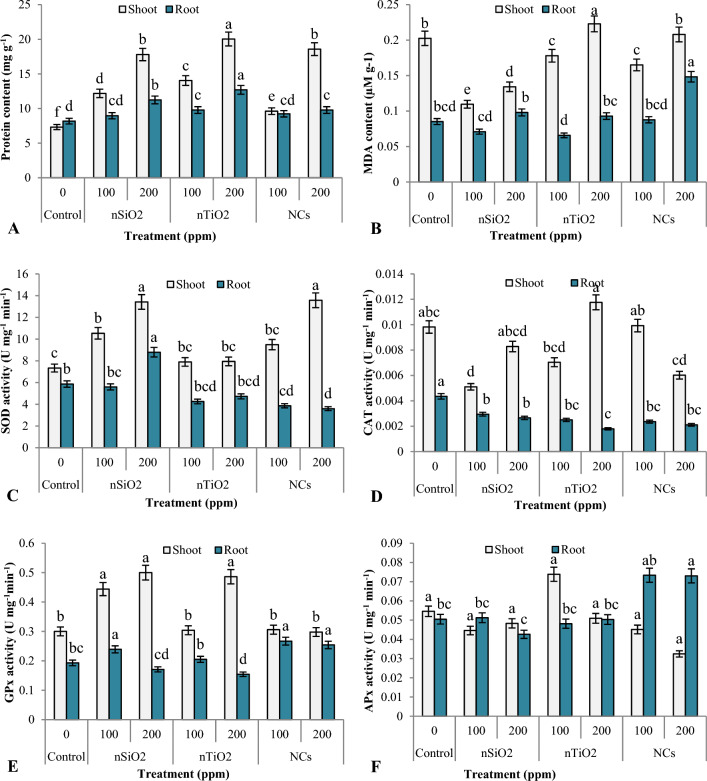
Protein (**A**) and MDA (**B**) contents, as well as the activities of SOD (**C**), CAT (**D**), GPx (**E**) and APx (**F**) in the shoots and roots of *Z. mays* treated with 100 and 200 ppm of nSiO_2_, nTiO_2_ and TiO_2_/SiO_2_ NCs. Data represents the mean of five replicates, with different letters denoting significant differences at *p* ≤ 0.05. Standard error bars indicate the variability of data.

Compared to the control group, both nanomaterials and NCs significantly increased protein content in both roots and shoots, with a more pronounced effect observed at 200 ppm compared to 100 ppm (Fig. 5A). Specifically, protein content increased by 66 and 143% in shoots and by 10 and 37% in roots at 100 and 200 ppm of nSiO_2_, respectively. For nTiO_2_, protein content increased by 92 and 174% in shoots and by 20 and 55% in roots at 100 and 200 ppm concentrations, respectively. Similarly, NCs led to a percentage increase of 31 and 13% at 100 ppm and 154 and 20% at 200 ppm concentration in shoots and roots of treated plants, respectively. Nanomaterials may modulate plant metabolism to trigger the synthesis of proteins involved in growth, development and stress responses^59^. Suriyaprabha et al.^37^ reported higher protein contents in maize following nSiO_2_ treatment. According to Yang et al.^56^, nTiO_2_ enhanced the absorption of inorganic N, as well as increased the activity of enzymes that help plants to synthesise protein and chlorophyll.

On the 30th day, the content of MDA in the shoots of all treated plants was lower than the control, except for nTiO_2_ at 200 ppm, where a 10% increase was observed (Fig. 5B). However, no appreciable difference in the MDA content of treated roots was observed. Reduced lipid peroxidation, which damages lipids and proteins in cells, is expected to be the source of higher protein concentrations in the treated plants. However, despite experiencing oxidative stress, the plants had the highest protein content at 200 ppm of nTiO_2,_ which correlate well with enhanced activities of antioxidant enzymes (SOD, CAT and GPx). Enhanced protein content is considered an adaptive response where the plants increase the synthesis of certain stress-related proteins involved in ROS detoxification^60^.

The nSiO_2_ are also well known for their ability to reduce oxidative stress, potentially through the scavenging of ROS and the upregulation of antioxidant enzyme activities (Fig. 5C–F). In the present work, nSiO_2_ raised the activities of shoot SOD (Fig. 5C) and GPx (Fig. 5E) at both 100 and 200 ppm. The CAT (Fig. 5D) and APx (Fig. 5F) activities decreased in shoots as well as in roots at both concentrations. Torabi et al.^61^ observed similar outcomes in *Borago officinalis* plants grown in the presence of Si and under salt stress. The authors observed that at 1.5 mM Si, the MDA levels decreased by 38%, whereas SOD activity increased significantly while the activity of CAT and APx remained unchanged.

The nTiO_2_ did not affect the shoot SOD and GPx activities but decreased the CAT activity at 100 ppm concentration, which correlated with a decrease in MDA content in the shoot of *Z. mays* plants, but at 200 ppm concentration of nTiO_2_, a slight increase in SOD, CAT and GPx activities and MDA content was observed. It indicated that nTiO_2_ developed stress at 200 ppm concentration in the shoot of *Z. mays* plants which was also evident from slightly reduced shoot growth at this concentration.

In roots, activities of all the antioxidant enzymes i.e., SOD, CAT, APx and GPx decreased at both the concentrations of nTiO_2_ which indicated that they did not harm the roots of *Z. mays* plants.

In the shoots of SiO_2_/TiO_2_ NCs treated *Z. mays* plants, SOD activity increased, APx activity decreased and GPx activity was not affected at both 100 and 200 ppm of NCs. The CAT activity increased at 100 ppm but decreased at 200 ppm of NCs. In roots, SiO_2_/TiO_2_ NCs decreased SOD and CAT activities and increased APx and GPx activities at both concentrations. Numerous factors, including the type and surface charge of the nanomaterial, its concentration, the duration of exposure and the specific plant species, might affect the intricate interaction between nanomaterials and antioxidant enzymes. Studies have revealed that the effects of nanomaterials on the activity of enzymes are inconsistent and unpredictable. For instance, nTiO_2_ enhanced the GPx, CAT, and SOD activities in *Lemna minor*^62^ and decreased the GR and APx activities in *Vicia faba*^63^. However, nTiO_2_ increased the activities of CAT, SOD, GPx, and APx in spinach^64^. Consequently, it remains challenging to make generalizations about the effects of nanomaterials on antioxidant enzymes.

### Influence on soil microorganisms and uptake of nutrients by plants

**Figure 6:**
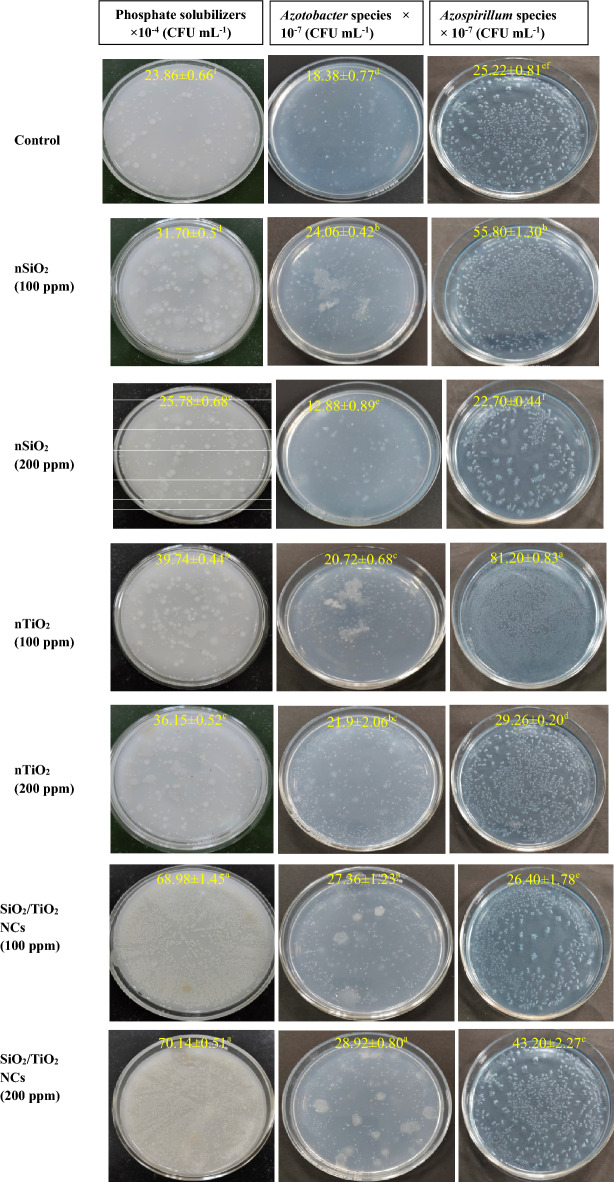
Activities of plant growth-promoting bacteria (CFU mL^−1^) as measured in the rhizospheric soil spiked with nSiO_2_, nTiO_2_ and SiO_2_/TiO_2_ NCs. Data is presented as mean ± S.D (n = 5), with different letters indicating significant differences at *p* < 0.05.

Plant productivity is highly influenced by soil microbes^65^. The impact of nanomaterials on plant development-promoting microorganisms, such as N-fixers and phosphate solubilizers, was investigated in the current study (Fig. 6).

Phosphate-solubilizing bacteria (PSB) can convert insoluble organic and inorganic P to soluble P that plants can easily absorb. P plays an important role in plant growth and development, N fixation and conversion of sugar to starch^66^. nSiO_2_ and nTiO_2_ treatment increased the population of PSB at both 100 and 200 ppm concentrations (Fig. 6). Due to the improved PSB count in the presence of both nanoparticles, higher P content in all the treated *Z. mays* plants was observed, except for reduced P content in the roots of nSiO_2_ treated plants (Table 3). While Si normally increases the bioavailability of nutrients in the soil, occasionally, particularly at higher concentrations and neutral pH, Si may immobilize P by forming complexes with phosphate ions or it may compete with P for uptake by plant roots^67^. When the soil was amended with SiO_2_/TiO_2_ NCs, PSB count was almost three times the control and much higher than recorded for the individual nanoparticles. Solubilization of phosphate by microorganisms correlated well with the uptake of P by *Z. mays* plants as given in Table 3.

**Table 3:** Nutrient uptake (N, P, Mg, K) by *Z. mays* from soil spiked with nSiO_2_, nTiO_2_ and SiO_2_/TiO_2_ NCs. Data is presented as mean (n = 5). Different letters on the values indicate significant differences at *p* < 0.05.

	Treatments	Concentrations (ppm)	Nitrogen (N)	Phosphorus (P)	Magnesium (Mg)	Potassium (K)
Shoot	Control	00	1.58 ± 0.005^e^	0.04 ± 0.002^ g^	0.11 ± 0.007f.	3.92 ± 0.007^ g^
	nSiO_2_	100	2.20 ± 0.0^b^	0.16 ± 0.0008^d^	0.11 ± 0.011f.	4.35 ± 0.0f.
		200	1.52 ± 0.01f.	0.08 ± 0.0004f.	0.30 ± 0.007^d^	6.12 ± 0.004^c^
	nTiO_2_	100	1.78 ± 0.01^d^	0.57 ± 0.004^b^	0.49 ± 0.01^b^	11.96 ± 0.008^a^
		200	1.45 ± 0.008^ g^	2.40 ± 0.01^a^	0.33 ± 0.01^c^	6.40 ± 0.004^b^
	NCs	100	2.12 ± 0.01^c^	0.09 ± 0.004^e0^	0.24 ± 0.01^e^	5.47 ± 0.004^e^
		200	4.07 ± 0.01^a^	0.29 ± 0.004^c^	0.58 ± 0.01^a^	5.81 ± 0.01^d^
Root	Control	00	1.29 ± 0.004^e^	0.24 ± 0.004^d^	0.13 ± 0.01^ g^	2.11 ± 0.008^e^
	nSiO_2_	100	2.47 ± 0.004^c^	0.18 ± 0.008^e^	0.25 ± 0.007^c^	5.31 ± 0.008^b^
		200	1.08 ± 0.004^ g^	0.09 ± 0.0008f.	0.21 ± 0.01^d^	2.56 ± 0.004^d^
	nTiO_2_	100	1.24 ± 0.008f.	0.27 ± 0.004^c^	0.31 ± 0.01^b^	8.13 ± 0.004^a^
		200	1.44 ± 0.004^d^	0.27 ± 0.004^c^	0.18 ± 0.01f.	2.63 ± 0.007^d^
	NCs	100	3.18 ± 0.004^b^	0.42 ± 0.004^a^	0.36 ± 0.004^a^	4.57 ± 0.004^c^
		200	4.29 ± 0.007^a^	0.28 ± 0.01^b^	0.20 ± 0.008^e^	7.56 ± 0.004^a^

Significant increase in the colonies of N-fixing bacteria, *Azospirillum* was observed at 100 ppm concentration of nSiO_2_ but the same decreased at 200 ppm of nSiO_2_, though the decrease was insignificant. The *Azospirillum* colonies increased at both concentrations of nTiO_2_ as compared to control but the enhancement was more at 100 ppm compared to 200 ppm. In the case of NCs, *Azospirillum* count increased in a concentration-dependent manner. The count of another N-fixing bacteria, *Azotobacter* increased after the nSiO_2_ treatment at 100 ppm but decreased significantly at 200 ppm. *Azotobacter* population increased in the presence of nTiO_2_ and NCs at both 100 and 200 ppm; the highest was at 200 ppm of NCs, which corresponded to the highest N uptake by plants treated with 200 ppm of SiO_2_/TiO_2_ NCs. Although the count of N-fixing bacteria increased at 200 ppm nTiO_2_, there was an approximately 8% decrease in the N content of the shoot compared to control plants. This discrepancy may be attributed to the generation of oxidative stress by nTiO_2_ at 200 ppm, which can influence N assimilation, storage and translocation within the plant potentially leading to changes in shoot N content. The combination of oxidative stress and low nitrogen content resulted in reduced shoot growth in the treated plants. For the rest of the treatment groups, an increase in the colonies of both N fixers correlated well with the data on N uptake by *Z. mays* plants. The nSiO_2_ at 200 ppm was not so conducive to the N fixers and the plants had the reduced N and lowest P contents amongst all the treated plants, whereas the NCs of SiO_2_ with TiO_2_ yield the highest PSB and *Azotobacter* activities corresponding to greater accumulation of N and P by plant shoot and roots.

Potassium (K) ranks as the third most important macronutrient, following N and P, required to enhance plant productivity. K enhances the utilization efficiency of N, which, in turn, is directly related to plant growth. Mg is another important micronutrient that plays a very important role in chlorophyll synthesis, carbon fixation and as a cofactor of various enzymes. Compared to the control plants, uptake of K and Mg by *Z. mays* plants increased in all treatment groups. The increase in the concentration of Mg was also supported by the increase in chlorophyll and carbohydrate content. The nanomaterials have greater surface area for mineral adsorption and reduce nutrient leaching which may enhance the bioavailability of mineral nutrients. Mali and Aert^68^ observed that in the presence of Si in the soil, nutrients like N, P and K were more available to the plants^69^. The nTiO_2_ may also improve the cation exchange capacity of the soil and help retain the nutrients in the root zone for plant uptake. Increased root growth in the presence of SiO_2_/TiO_2_ NCs along with their unique surface properties and catalytic activity may have promoted nutrient retention and transport in the rhizosphere, improving their uptake by plants.

## Conclusion

**Figure 7:**
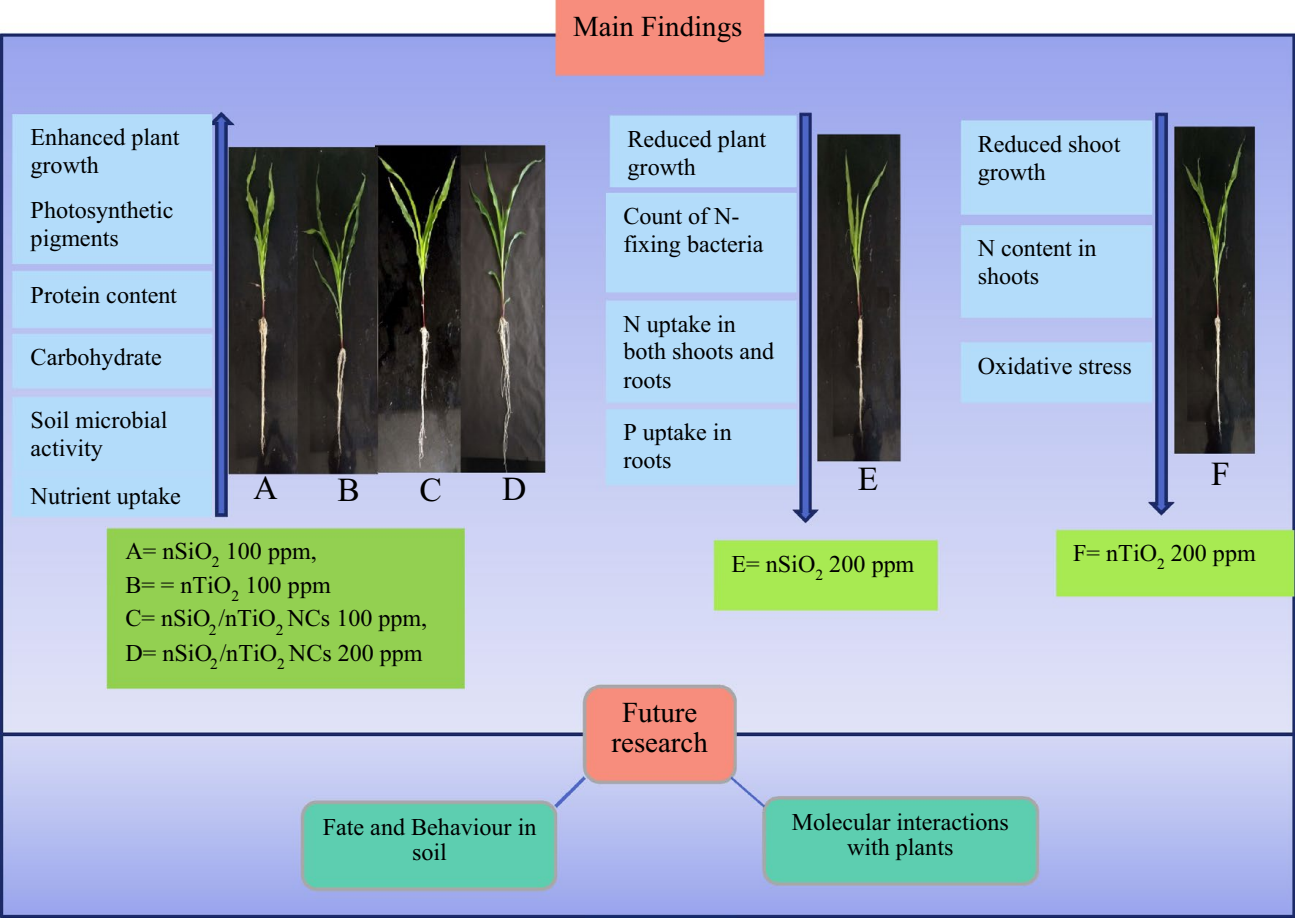
Effect of nSiO_2_, nTiO_2_, and SiO_2_/TiO_2_ NCs on *Z. mays* growth and soil health and future research requirements for their sustainable utilization in agriculture.

Considering the substantial impact of nanomaterials and NCs on plant growth and soil health, the effect of adding nSiO_2_, nTiO_2_ and SiO_2_/TiO_2_ NCs to the soil at 100 and 200 ppm concentrations has been described on *Z. mays* health, soil microbial activity and nutrient uptake by plants (Fig. 7). The results on plant growth, chlorophyll levels, photosynthetic and transpiration rate, stomatal conductance, as well as protein, carbohydrates and MDA levels, soil microbial activity and plant nutrient (N, P, Mg and K) uptake along with changes in the activities of antioxidant enzymes, suggest that these nanomaterials can be safely used to boost soil fertility and support *Z. mays* growth. However, nSiO_2_ and nTiO_2_ at 200 ppm concentration had adverse effects. nSiO_2_ at 200 ppm decreased shoot and root length, the count of N-fixing bacteria, N uptake in both shoots and roots and P uptake in roots, while increasing the root MDA contents. Decreased shoot growth in plants exposed to 200 ppm of nTiO_2_ was attributed to reduced N contents in the shoots and oxidative stress induced by the nanoparticles. In contrast, SiO_2_/TiO_2_ NCs at 200 ppm resulted in the maximum stimulation of plant growth, surpassing both nSiO_2_ and nTiO_2_ used individually at the same concentration, highlighting the superiority of NCs. The main findings of the study are given in Fig. 7.

Nevertheless, there is a need for further research on the fate, behaviour and transformation of nanomaterials in soil, their interaction with soil organic matter and their effects on other soil microorganisms. Additionally, the intricate molecular-level interactions of nanomaterials with plants remain an evolving area of research and a comprehensive understanding of these interactions is essential for the sustainable use of nanomaterials in agriculture.

### Supplementary Information

Supplementary Figure 1.

## Data Availability

All data generated during study has been included in the manuscript.
