# Supplementary material for: Unravelling the effects of nano SiO2, nano TiO2 and their nanocomposites on Zea mays L. growth and soil health
Source: Sci Rep. 2024 Jun 18;14:13996. doi: 10.1038/s41598-024-61456-x (PMC11183139; doi:10.1038/s41598-024-61456-x)
Supplement: Supplementary file 1 — Supplementary Figure 1. [file 41598_2024_61456_MOESM1_ESM.docx]

**256.6**

**B**

**D**

**C**

**A**

0

100000

200000

300000

400000

-100

0

100

200

**Total Counts**

**Apparent Zeta Potential (mV)**

0

1

2

3

4

5

6

7

8

0.1

1

10

100

1000

10000

**Intensity (Percent)**

**Size (d.nm)**

**-25.2**

0

5

10

15

20

0.1

1

10

100

1000

10000

**Intensity (Percent)**

**Size (d.nm)**

0

100000

200000

300000

400000

500000

600000

-100

0

100

200

**Total Counts**

**Apparent Zeta Potential (mV)**

**369.1**

**38.2**

**F**

**E**

0

2

4

6

8

10

12

14

16

0.1

1

10

100

1000

10000

**Intensity (Percent)**

**Size (d.nm)**

**4.09**

**375.5**

0

100000

200000

300000

400000

500000

-100

0

100

200

**Total Counts**

**Apparent Zeta Potential (mV)**

**Supplementary figure S1:** Zeta average size distribution and zeta potential of nSiO_2_ (A and B), nTiO_2_ (C and D) and SiO_2_/TiO_2_ NCs (E and F), respectively. The plot area represents the average hydrodynamic diameter (d.nm) of nanomaterials
